# Functional importance of Asp264 in the ketosynthase domain of Pks13 in *Mycobacterium smegmatis*

**DOI:** 10.1016/j.tcsw.2026.100178

**Published:** 2026-06-24

**Authors:** Mamata Modak, Hemza Ghadbane, Klaus Futterer, Apoorva Bhatt, Gurdyal S. Besra

**Affiliations:** School of Biosciences, Institute of Microbiology and Infection, University of Birmingham, Edgbaston, Birmingham B15 2TT, United Kingdom of Great Britain and Northern Ireland

**Keywords:** Mycolic acids, Mycobacterium, Pks13, Cell wall

## Abstract

Mycolic acids are key components of the mycobacterial cell envelope, contributing to its structural integrity and intrinsic resistance to environmental stress. The polyketide synthase Pks13 catalyzes the final Claisen condensation step in mycolic acid biosynthesis and is conserved across *Corynebacteriales*. While the catalytic cysteine within the ketosynthase (KS) domain is established as essential for activity, the role of surrounding residues in supporting catalysis remains less well understood. Herein, we utilized a *Mycobacterium smegmatis* conditional *pks13* mutant, and subsequent complemented strains harbouring a plasmid-borne copy of *pks13* to probe the functional importance of selected residues within the KS domain from previous structural studies. These site-directed mutagenesis studies identified Asp264 as critical for Pks13 function. The D264A strain showed severely impaired growth along with a pronounced loss of mycolate synthesis and mycolate-containing lipids: trehalose dimycolate (TDM) and trehalose monomycolate (TMM). In contrast, other mutations had either little or no significant effect on cell viability or mycolate synthesis or mycolate-containing lipids. Structural modelling of the KS domain suggests that loss of Asp264 disrupts the local active-site environment, resulting in compaction of the binding pocket and altered conformation of a loop proximal to the catalytic residue Cys267. Together, these findings demonstrate that Asp264 is essential for Pks13 function and likely contributes to maintaining the structural environment of the KS domain.

## 1 Introduction

The mycobacterial cell envelope is a complex, multi-layered structure composed of peptidoglycan, arabinogalactan, a mycolic acid-rich layer, and an outer capsule. This architecture is essential for bacterial survival under diverse and harsh environmental conditions (Daffé and Marrakchi, 2019). A key feature of mycobacteria and corynebacteria is their lipid-rich cell wall, largely due to the presence of long-chain mycolic acids, linked to intrinsic drug resistance in mycobacteria (Hart and Bernhardt, 2025; Jackson, 2014). These lipids play an important role in determining cell envelope properties, including permeability (Bansal-Mutalik and Nikaido, 2014), biofilm formation in *Mycobacterium tuberculosis* (Ojha et al., 2005), and pathogenicity (Barry III et al., 1998; Daffé and Draper, 1997; Dubnau et al., 2000; Vander Beken et al., 2011).

**Fig. 1:**
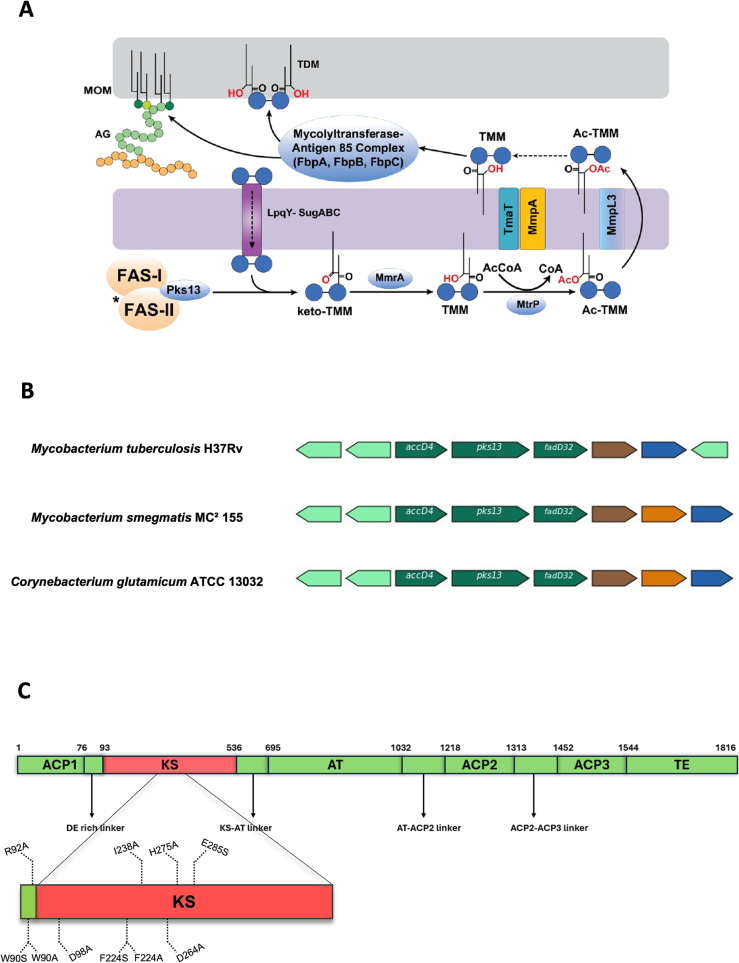
**Terminal stages of mycolic acid biosynthesis and the role of Pks13.** A) Simplified scheme depicting mycolic acid biosynthesis: FAS-I and FAS-II synthesize fatty acid precursors that are condensed by Pks13 to form keto-trehalose monomycolate (TMM). Sequential reduction by MmrA yields TMM and acetylation by MtrP to yield Ac-TMM prior to transport. Ac-TMM is flipped across the cytoplasmic membrane by the MmpL7 transporter, assisted by the membrane proteins TmaT and MmpA. The LpqY-SugABC ABC transporter recycles extracellular TMM back into the cell. In the periplasm, Ac-TMM is deacetylated to TMM, which serves as substrate for the Antigen 85 complex, transferring mycolic acids to arabinogalactan (AG) or to another TMM to form trehalose dimycolate (TDM) in the mycomembrane. B) Conservation of *accD4-pks13-fadD32* gene cluster in *M. tuberculosis*, *M. smegmatis* and *C. glutamicum*. Genes are shown as arrows indicating orientation. This cluster encodes key enzymes involved in mycolic acid biosynthesis. Genes are coloured according to homology. The genes which show no homology are coloured in light green. C) Schematic representation of the *pks13* gene from *M. smegmatis*. Key catalytic domains including acyl carrier protein (ACP), ketosynthase (KS), acyltransferase (AT) and thioesterase (TE) are indicated. The KS domain is enlarged below to show the positions of the site-direction mutations generated in this study. (For interpretation of the references to colour in this figure legend, the reader is referred to the web version of this article.)

Consistent with its essential role, Pks13 is highly conserved across members of the order *Corynebacteriales*, including *Mycobacterium smegmatis*, *M. tuberculosis* and *Corynebacterium glutamicum* (Bon et al., 2022), and catalyzes a late-step in mycolic acid biosynthesis, with key accessory enzymes and substrates (Gande et al., 2004; Portevin et al., 2004; Portevin et al., 2005; Yamaryo-Botte et al., 2015) (Fig. 1a, b). Pks13 resembles a large multi-domain Type I polyketide synthase, composed of sequential catalytic domains, including acyl carrier protein (ACP), ketosynthase (KS), acyltransferase (AT), and thioesterase (TE) domains (Fig. 1c). These domains function in a coordinated manner to mediate the transfer and condensation of two long-chain fatty acid substrates with the KS domain forming the central catalytic site for chain elongation. The meromycolate chain, synthesized by the fatty acid synthase type II (FAS-II) elongation system is activated by an fatty acyl-AMP ligase, FadD32 and loaded onto the N-terminal ACP domain of Pks13; this constitutes the initiation of mycolic acid Claisen condensation (Gavalda et al., 2009). Concurrently, the FAS-I derived α-branch fatty acid is carboxylated by AccD4 containing acyl-CoA carboxylase complex (Portevin et al., 2005). Substrates are shuttled between active sites ultimately leading to the formation of a mycolic acid precursor that is released *via* the C-terminal TE domain (Kim et al., 2023). In addition to this canonical PKS architecture, Pks13 has been shown to harbour acyltransferase activity, suggesting it plays a broader enzymatic role in mycolic acid biosynthesis (Gavalda et al., 2014).

Indeed, recent structural studies indicate that the enzyme retains a canonical polyketide synthase architecture, reflecting strong evolutionary conservation of its catalytic framework, while incorporating adaptations that enable the processing of long-chain mycolic acid substrates (Johnston et al., 2024; Kim et al., 2023). The KS domain is one of the most conserved catalytic modules within Type I polyketide synthases with sequence conservation observed across diverse bacterial species. Several of these conserved residues are in close proximity to the active site cysteine and are essential for catalytic activity, highlighting the strong evolutionary constraints acting on the KS domain and its catalytic machinery (Robbins et al., 2016). In addition, the gene cluster comprising *fadD32–pks13–accD4* which encodes key components required for the Claisen condensation step of mycolic acid biosynthesis are conserved across *Corynebacterineae* (Fig. 1b) with high sequence identity (Kim et al., 2023). Importantly, loss of Pks13 results in complete absence of mycolic acids, leading to severe defects in cell envelope integrity and cell viability (Xia et al., 2023). This highlights Pks13 as an essential enzyme and potential drug target.

Structural studies have shown that Pks13 functions as a homodimer with the KS domain positioned centrally and contributing to dimerization, while the other domains extend outward from this core (Johnston et al., 2024). Within the KS domain, a conserved cysteine residue (C267 in *M. smegmatis*) is essential for activity and sits at the base of a hydrophobic channel that accommodates the meromycolate chain. Structural studies also indicate that efficient substrate positioning depends not only on this catalytic residue but also on the surrounding active-site architecture (Kim et al., 2023). Whilst, C267 is well defined as the catalytic nucleophile, the role of neighbouring residues in maintaining functional integrity of the KS domain is still not well understood.

In this study, we have used a conditional *M. smegmatis pks13* mutant complemented with a functional copy of *pks13*, along with a set of site-directed mutants targeting residues within and around the KS domain to investigate their role in enzyme function and mycolic acid synthesis. From the panel, the D264A variant showed a pronounced phenotype, severely impaired growth and a marked loss in mycolic acids and other cell envelope mycolate-containing lipids.

## 2 Materials and methods

### 2.1 Bacterial strains and culture conditions

*M. smegmatis* strains used in this study include a conditional ∆*pks13* mutant, complemented strains with wild type *pks13* and a panel of site-directed mutants (SDM). The ∆*pks13* mutant was grown in tryptic soy broth (TSB) supplemented with hygromycin (100 μg/mL), kanamycin (30 μg/mL), and Tween-80 (0.05%). The complemented and SDM strains were grown in TSB supplemented with hygromycin (100 μg/mL), kanamycin (30 μg/mL), apramycin (50 μg/mL) and Tween-80 (0.05%). For induction of *pks13* expression, acetamide was added at a final concentration 0.2%. All cultures were incubated at 37 °C for 24–48 h, unless otherwise specified.

### 2.2 Generation of the *M. smegmatis* conditional ∆*pks13* mutant

The rescue (complementing) plasmid containing *M. tuberculosis pks13* was PCR amplified from genomic DNA with a BamHI restriction site incorporated into the primers (Table 1). The amplified fragment and the *Escherichia coli*/mycobacterial shuttle vector pSD26 (Daugelat et al., 2003) were both digested with BamHI, followed by ligation using the T4 DNA ligase to generate the recombinant plasmid pSD26-Mt-*pks13*. This construct contains the Mt-*pks13* gene flanked by the mycobacterial acetamidase promoter and a hygromycin resistance cassette. In addition, the construct includes a sequence encoding a C-terminal hexahistidine (His₆) tag fused to Mt-*pks13*.

**Table 1:** Oligonucleotides used for the construction of *M. smegmatis pks13* conditional mutant.

Name	DNA Sequence (5′- 3′)
**PCR amplification of *M. tuberculosis pks13***	
uppks	GATCGATCGGATCCATGGCTGACGTAGCGGATT
dnpks	GATCGATCGGATCCCTGCTTGCCTACCTCACTG
**Site-directed mutagenesis at HindIII site**	
sdm	CGCTTCAAAGCTCTCTAGCAGAAAT
**Construction Δ*M. smegmatis pks13***	
UP5	GATCGATCACTAGTTGTGCCGAAGCCGGGCTCAC
UP3	GATCGATCGATATCATGTGCGCAGCCACTCCCGC
DN5	GATCGATCTCTAGAAGGCGCTCAACCGCATCGGGCTC
DN3	GATCGATCCTTAAGTCGCGCCCGACAACACCAT

To generate a merodiploid strain expressing both *M. smegmatis* and *M. tuberculosis* copies of *pks13*, the acetamidase promoter together with the His₆-tagged Mt-*pks13* sequence were sub-cloned into the mycobacterial integrative plasmid pMV306 (Kan^R^) (Murry et al., 2005) prior to the disruption of the native *M. smegmatis pks13*.

To facilitate the transfer of the expression cassette, an *Xba*I – *Hin*dIII fragment from the pSD26-*pks13* was targeted. However, the presence of an internal HindIII restriction site within the acetamidase promoter necessitated its removal by site-directed mutagenesis. This modification generated a new construct, pSD26a-Mt-*pks13*. The new construct was double-digested with *Xba*I and HindIII to isolate the Ac-Mt-*pks13*-His₆ fragment, which was then ligated into the similarly digested pMV306 to yield the construct pHG-Mt-*pks13*. This was electroporated into *M. smegmatis* competent cells resulting in the generation of *M. smegmatis*::pHG-Mt-*pks13*.

The *M. smegmatis*∆*pks13* mutant was generated using specialized transduction as described previously (Bardarov et al., 2002; Jain et al., 2014). For the knockout of the native *M. smegmatis pks13* gene in *M. smegmatis*::pHG-Mt-*pks13*, approximately 1 kb regions upstream and downstream of the *M. smegmatis pks13* gene were PCR amplified to generate homologous flanking sequences for allelic exchange. The upstream fragment was digested with *Spe*I and *Eco*RV, while the downstream fragment was digested with XbaI and *Sma*I. These fragments were sequentially ligated into the allelic exchange vector pJSC347, which carries the λ phage cos site and a hygromycin resistance marker. This results in a construct pJSC347–Ms-*pks13* in which the flanking regions are surrounded by a hygromycin resistance cassette. This recombinant plasmid is *Pac*I-digested and ligated into PacI digested temperature-sensitive mycobacteriophage phAE159. Ligation products were packaged and used to infect *E. coli* HB101 to recover recombinant phasmids. These were confirmed by PacI digestion. High-titre phage lysates were prepared by infecting *M. smegmatis* overlaying infected cultures in top agar and harvesting plaques after incubation at 30 °C. Phage lysates were filtered and titrated to obtain stocks ≥10^10^ PFU /mL. Specialized transduction was performed by incubating washed mid-log phase mycobacterial cells with high-titre recombinant phage at the non-permissive temperature (37 °C) to allow allelic exchange without further phage replication Following incubation, the culture was centrifuged and washed twice to remove residual phage. The final cell pellet was resuspended in TSB, and cells were plated on hygromycin, kanamycin and acetamide containing agar to select recombinant clones. Resistant colonies were screened to confirm gene replacement using whole genome sequencing and PCR, yielding the desired deletion mutant.

### 2.3 Generation of the *M. smegmatis*∆*pks13* mutant and complementing Ms-*pks13* variants

The *M. smegmatis*∆*pks13* mutant carries the *M. tuberculosis pks13* gene under control of an acetamide inducible promoter. To further complement this conditional strain, the *M. smegmatis pks13* gene was PCR amplified (Table 2) and cloned into the *E. coli*/mycobacterial shuttle vector pMV261, a constitutive expression vector under the control of a hsp60 promoter, which continuously expresses the gene without the presence of an inducer (Stover et al., 1991). The amplified fragment and the vector were both digested with *Hin*dIII and subsequently ligated with T4 DNA ligase. pMV261 carries kanamycin resistance, since the *M. smegmatis* conditional *pks13* mutant strain also carries a kanamycin resistance, a modified version of pMV261 carrying an apramycin resistance was used. Following transformation into *E. coli* cells, the recombinant colonies were selected on apramycin containing agar plates. Resistant clones were screened and confirmed by sequencing. The resulting complemented plasmid construct consists of pMV261-Apr – Ms*-pks13*. The complemented plasmid was electroporated into the *M. smegmatis∆pks13* strain. Following recovery, transformants were selected on agar plates containing hygromycin, kanamycin, apramycin and acetamide to confirm successful complementation.

**Table 2:** Oligonucleotides used for the complementation of the *M. smegmatis* conditional *pks13* mutant.

Name	DNA Sequence (5′- 3′)
Ms-Pks13 FP	CCAAAAGCTTATATGACCGTCAACGA
Ms-Pks13 RP	TTACAAGCTTTCACTTGGCCCCATCCTC

### 2.4 Generation of site-directed mutants of the KS domain

Single point mutations were introduced to the pMV261 Apr–*M. smegmatis pks13* plasmid using PCR-based site-directed mutagenesis using the Q5 High-Fidelity DNA Polymerase system (Table 3). The mutagenic primers designed are listed in Table 4. Primers contained the mutation centrally, flanked by complementary sequences to ensure efficient amplification. PCR amplification was performed under Hot Start conditions to improve specificity and reduce non-specific amplification. PCR reactions were prepared in a total volume of 25 μL. Amplification was performed under the following conditions: initial denaturation at 98 °C, 1 min; 25 cycles of denaturation at 98 °C, 30 s, annealing at 69 °C for 30 s and extension at 72 °C for 11 min; followed by a final extension at 72 °C for 10 min. Amplified products were subjected to Kinase-ligase-*Dpn*I (KLD) treatment to circularize the PCR product and remove parental template DNA (D'Ambrosio et al., 2022). Reactions were incubated at room temperature for 1 to 1.5 h. The entire KLD reaction was used to transform 100 μL of *E. coli* competent cells. All constructs were verified by sequencing to confirm the presence of desired mutations. The confirmed mutant plasmids were then electroporated into the *M. smegmatis*∆*pks13* strain for subsequent phenotypic and functional analysis. Electrocompetent cells were prepared according to standard protocols (Parish, 2021). Briefly, cells were grown to mid-log phase, washed extensively with ice-cold 15% glycerol and concentrated prior to transformation. Approximately, 1 μg of plasmid DNA was mixed with 300 μL of competent cells and transferred to a pre-chilled electroporation cuvette. Electroporation was performed at 2500 eV. Following pulsing, cells were recovered in 2 mL of TSB and incubated with shaking for 6 h at 37 °C. Recovered cells were plated on TSB plates with appropriate antibiotics and incubated at 37 °C for 48 h. Transformants were screened to confirm the presence of the mutant plasmid and selected strains were used for subsequent analysis. The presence of *pks13* gene among the *M. smegmatis* site-directed mutants was confirmed using RT-PCR (Supplementary Fig. 1).

**Table 3:** Residues selected for mutagenesis and functional studies.

Residue	Type	Rationale
W90, F224	Aromatic	To assess their role in forming and maintaining the hydrophobic substrate channel
D98, D264, E285	Charged (acidic)	To assess their contribution to structural stability and local electrostatic interactions
R92	Charged (basic)	To assess their contribution to structural stability and local electrostatic interactions
I238	Hydrophobic	To assess contribution to overall protein folding and KS structural integrity
H275	Polar	To evaluate role in maintaining active-site loop conformation and geometry

**Table 4:** Oligonucleotides used to generate single point mutations in this study.

Name	DNA Sequence (5′- 3′)
D264A FP	CGTGGCGGTCGCGACCGCGTGCTC
D264A RP	GACGGGCCGCGGAAGTCGTA
W90A FP	GGACGAGGACGCGTCGCGCACACGC
W90A RP	GCGTGTGCGCGACGCGTCCTCGTCC
W90S FP	GGACGAGGACAGCTCGCGCACACGC
W90S RP	TCGTCGTACGGCTCGGGCTC
R92A FP	GGACTGGTCGGCGACACGCGATGTCGAGGAC
R92A RP	TCGTCCTCGTCGTACGGCTC
F224S FP	CGATTACAGCAGCCTGGCGATGAGC
F224S RP	TTGGTCGAGCTGCCGATGTA
F224A FP	CGATTACAGCGCGCTGGCGATGAGCGATC
F224A RP	TTGGTCGAGCTGCCGATGTA
D98A FP	CGATGTCGAGGCGATCGCGATCGTCGG
D98A RP	CGTGTGCGCGACCAGTCCTC
E285S FP	GCGCGCCGGTAGCGCCGACGTCG
E285S RP	AGTGCCTGCACACCCTGGTG
I238A FP	TCCCTACGCCGCGACCGGCACCGC
I238A RP	TGCGCGATCGACGGATCGCT
H275A FP	GGTCGCCACGGCGCAGGGTGTGCAG
H275A RP	AGCGAGCTGGAGCACGCGGT

### 2.5 RNA extraction and RT-PCR

To confirm transcription of plasmid borne *Ms pks13* in the complemented strain and site-directed mutant strains, RT-PCR was performed. 100 mL of bacterial cultures were grown to mid log phase in TSB broth supplemented with appropriate antibiotics. Total RNA was extracted using the Direct-zol RNA miniprep kit (Zymo Research) according to the manufacturer's instructions. Residual genomic DNA was removed by DNase I treatment followed incubation at room temperature. RNA integrity and concentration was assessed by NanoDrop. First-stand cDNA was synthesized from 500 ng total RNA using the iScript cDNA synthesis kit (Bio-Rad) according to manufacturer's instructions which employs a blend of oligo and random hexamer primers. A no reverse transcriptase control was included for each sample to confirm absence of genomic DNA contamination.

PCR amplification was performed using *M. smegmatis* specific *pks13* primers (Table 5) to detect transcription of plasmid encoded *Ms pks13* copy since the rescue plasmid of the conditional mutant has the *M. tuberculosis pks13* gene. The housekeeping *sigA* was amplified in parallel as a loading and quality control. PCR reactions were carried out using Q5 polymerase. PCR products were resolved by agarose gel electrophoresis on a 2% gel and visualized under UV illumination.

**Table 5:** Oligonucleotides used for the RT-PCR.

Name	DNA Sequence (5′- 3′)
**Pks13 (*M. smegmatis*)**	
FP	ATGACCGTCAACGAGATGCG
RP	CGTGTGCGCGACCAGTCCTCGTC
**SigA (*M. smegmatis*)**	
FP	GTGGCAGCGACAAAGGCAAG
RP	TCTCGCCCTTCTCGGCGAGTTC
**Pks13 (*M. tuberculosis*)**	
FP	ATGGCTGACGTAGCGGAATCC
RP	ATGATCCGGGTGGCCAGC

### 2.6 Growth analysis and colony forming unit assay

Growth patterns between the *M. smegmatis* conditional *pks13* mutant, wild type *pks13* complemented strain, and SDM strains, were assessed in liquid broth and by colony forming unit (CFU) on solid agar under both inducing and non-inducing conditions. Primary cultures were initiated from single colonies in TSB and grown under inducing conditions. Liquid cultures were then washed three times with fresh TSB to remove residual acetamide and used to inoculate fresh media under both inducing and non-inducing acetamide (+Ac and —Ac) conditions, respectively, maintaining the same antibiotic concentrations as mentioned earlier. For solid agar CFU determination, cultures grown for 24 h under inducing and non-inducing conditions were mixed and serially diluted up to 10^−8^ in sterile TSB. Each dilution aliquot (5 μL) was spotted onto TSB agar plates containing the appropriate antibiotics and either supplemented with acetamide or without acetamide to maintain inducing and non-inducing conditions, respectively. Plates were incubated for 3 days at 37 °C. CFU were assessed based on visible colony growth and growth differences were compared under both conditions.

### 2.7 Extraction and analysis of [^14^C]-labelled total mycolates and mycolate-containing lipids

To investigate the impact of the *pks13* site-directed mutations on lipid biosynthesis, metabolic labelling with [^14^C]-acetate was performed using *M. smegmatis* cultures. Cultures were initially grown on TSB agar plates supplemented with appropriate antibiotics and acetamide at 37 °C for 2–3 days. Single colonies were used to inoculate a 5 mL TSB broth culture supplemented with appropriate antibiotics, acetamide, and incubated further at 37 °C with shaking, this is the primary culture. Acetamide was supplemented to all *M. smegmatis* cultures throughout the primary culture phase to maintain expression of Pks13. Once the primary cultures reached an OD 600 nm ∼ 0.8, cells were harvested by centrifugation and washed with TSB to remove residual acetamide. The resulting pellets were resuspended in TSB alone, 1 mL of the primary culture was transferred into two 10 mL cultures: one supplemented with acetamide and one without. Radiolabelling was initiated at the time of inoculation by the addition of 5 μCi/mL [^14^C]-acetate. Cultures were incubated for a further 24–48 h at 37 °C under shaking conditions. The cells were then harvested by centrifugation (4000 rpm, 10 min), washed once with PBS, and dried either under a gentle stream of nitrogen or using a vacuum concentrator.

For detailed analysis of total fatty acid and mycolic acid composition, fatty acid methyl esters (FAMEs) and mycolic acid methyl esters (MAMEs) were prepared using tetra-butyl ammonium hydroxide (TBAH)-mediated hydrolysis and methylation using methyl iodide according to established methods (Butler et al., 1991; Garton et al., 2002). The resulting FAMEs and MAMEs (20,000 cpm) were resolved by thin-layer chromatography (TLC) on Silica Gel 60 F254 plates (Merck) using petroleum-ether:acetone (95:5 × 3). Total lipids were extracted following established protocols as described previously (Dobson et al., 1985; Minnikin et al., 1982), and samples (20,000 cpm) analyzed by TLC on Silica Gel 60 F254 plates (Merck) using chloroform: methanol: ammonium hydroxide (80:20:2, *v*/v/v), to resolve trehalose dimycolate (TDM) and trehalose monomycolate (TMM). Radiolabelled FAMEs, MAMEs, and mycolate-containing lipids were visualized by autoradiography following exposure to Kodak X-Omat AR film for 4–5 days.

## 3 Results and discussion

### 3.1 Generation of *M. smegmatis*∆*pks13* mutant

**Fig. 2:**
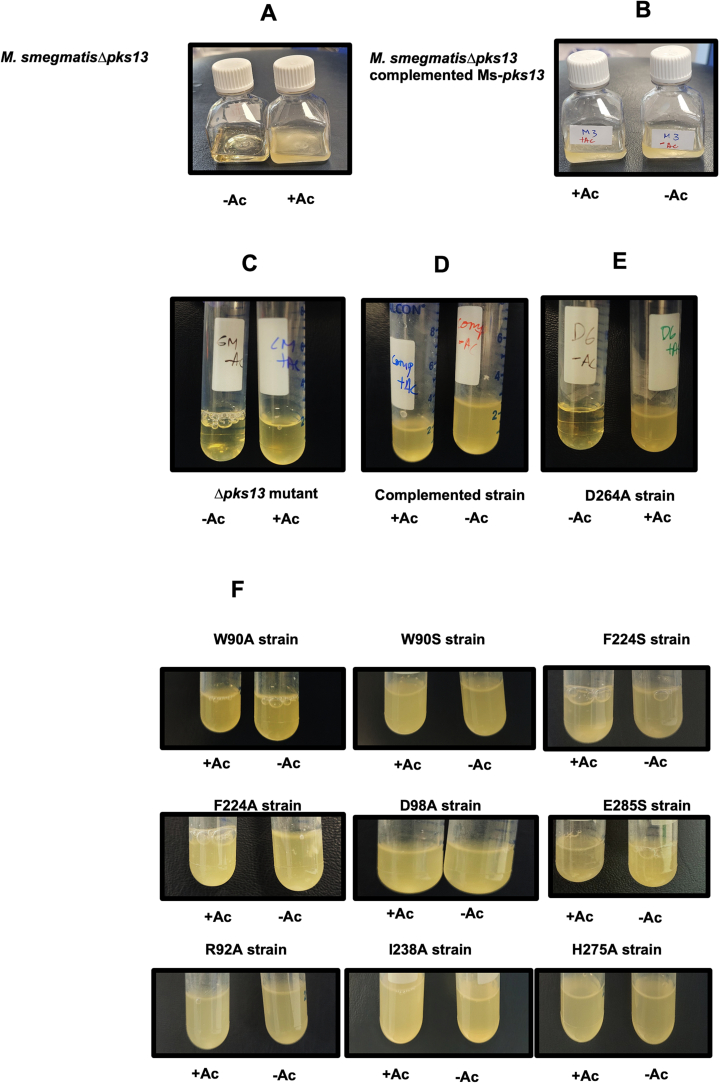
**Complementation of the *M. smegmatis*∆*pks13* under inducing and non-inducing conditions of acetamide (Ac) demonstrating growth restoration in liquid culture.** A) ∆*pks13* mutant. B) complemented ∆*pks13* strain and wild type Ms*-pks13*. C) ∆*pks13* mutant. D) complemented ∆*pks13* strain. E) ∆*pks13* D264A. E) A panel of ∆*pks13* strains complemented with SDMs (Table 4).

The *M. smegmatis*∆*pks13* mutant was generated using specialized transduction (Bardarov et al., 2002; Jain et al., 2014) using a rescue plasmid containing *M. tuberculosis pks13*. The conditional mutant was only able to grow in the presence of acetamide (Fig. 2a). The restoration of growth demonstrates functional complementation of the essential *pks13* gene. These results confirm that the observed growth defect of the ∆*pks13* mutant in non-inducing conditions is specifically due to the depletion of the Pks13 activity. Following successful rescue of growth, an pMV261Apr - *M. smegmatis pks13* construct was used as a backbone for site-directed mutagenesis to investigate the functional role of KS domain of Pks13 within ∆*pks13* within key residues selected for this study shown in Table 3.

### 3.2 Growth characterization of *M. smegmatis∆pks13* strains

Following complementation using pMV261Apr – *M. smegmatis pks13* plasmids (Fig. 2b), whole cell growth of ten site-directed mutants was evaluated in both liquid culture and solid-agar by CFU analysis. In liquid culture, the conditional ∆*pks13* mutant (Fig. 2a,c) shows growth in only +Ac conditions suggesting near native levels of *pks13* expression as shown by RT-PCR (Supplementary Fig. 1); the complemented strain (Fig. 2b, d) shows growth in both presence and absence of Ac, however the D264A mutant shows pronounced reduction in turbidity compared to the +Ac condition suggesting loss of viability in the absence of inducer (Fig. 2e). In contrast, the other mutants did not show any significant difference between +Ac and —Ac conditions in liquid broth (Fig. 2f). For CFU determination, samples were collected after 24 h and serially diluted up to 10^−8^. Appropriate dilutions were plated on TSB agar containing hygromycin (100 μg/mL), kanamycin (30 μg/mL), apramycin (50 μg/mL) with or without acetamide (0.2%) to compare growth under inducing and non-inducing conditions.

**Fig. 3:**
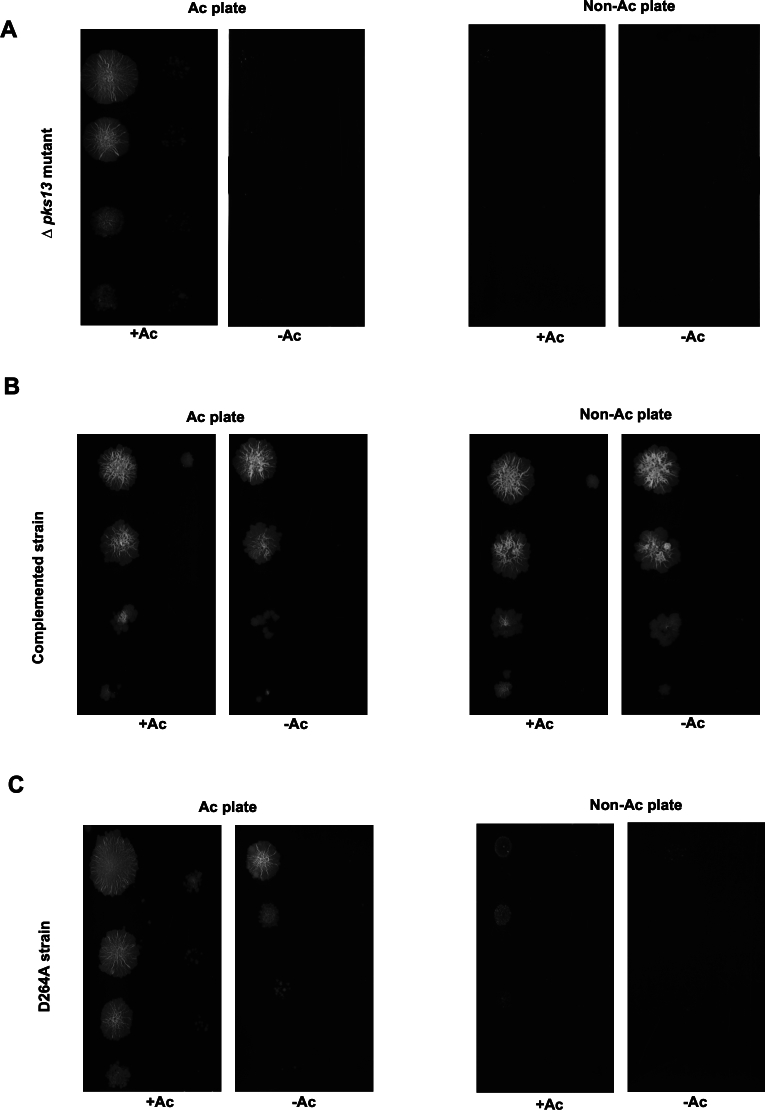
**Serial dilution assay of A) ∆*pks13* mutant B) complemented ∆*pks13* strain C) ∆pks13 D264A.** The +Ac and —Ac cultures were grown on both Ac plates and non-Ac plates.

CFU analysis of the ∆*pks13* mutant shows severe growth defect in the absence of acetamide (Fig. 3a), whilst the complemented strain (Fig. 3b) shows growth in the presence and absence acetamide. Consistently, there is a severe growth defect observed in the D264A mutant (Fig. 3c). On acetamide containing plates, cultures grown under inducing conditions (+Ac) showed robust growth with colonies observed up to a 10^−7^ dilution (10^−6^ = 9 colonies; 10^−7^ = 8 colonies). In contrast, cultures grown without acetamide displayed a sharp reduction in viability, with colony formation dropping significantly beyond a 10^−3^ dilution (10^−3^ = 11 colonies; 10^−4^ = 3; 10^−5^ = 2; 10^−6^ = 1). When plated on non-acetamide plates, the acetamide culture showed limited growth only up to a 10^−3^ dilution, whilst the culture in absence of acetamide exhibited minimal to no detectable growth. Together, these observations indicate that the D264 residue is critical for Pks13 function and its substitution leads to loss of function and failure to rescue the conditional mutant. This highlights the essential role of D264 in maintaining normal growth and survival.

### 3.3 Lipid analysis of *M. smegmatis*∆*pks13* mutant and site-directed mutant strains

**Fig. 4:**
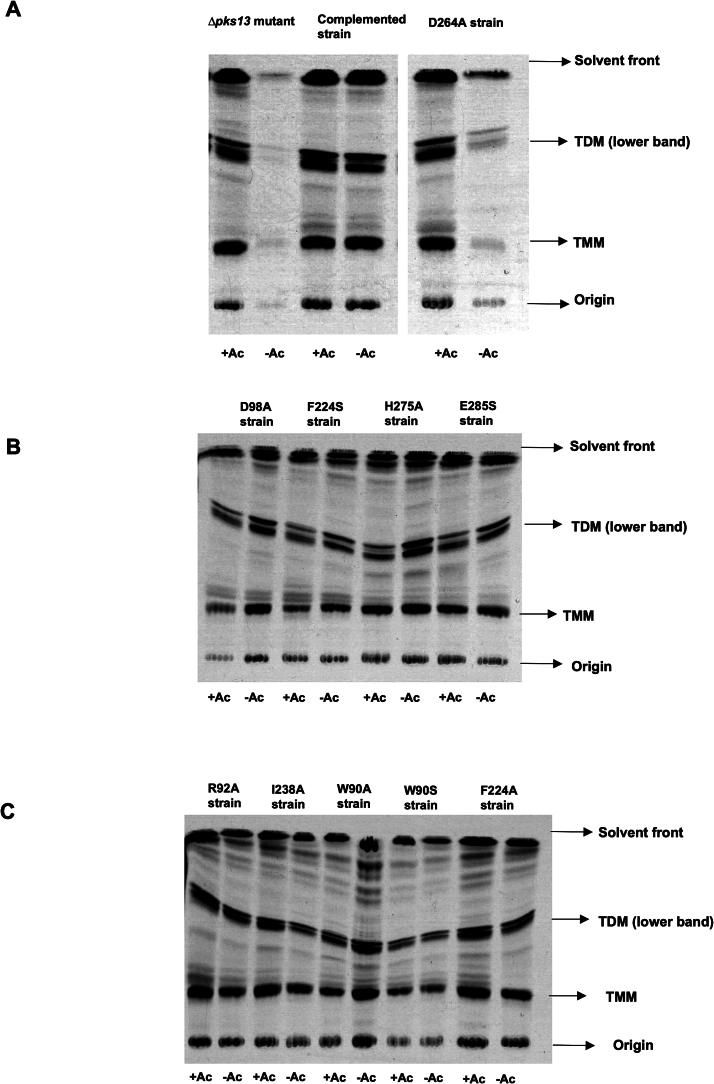
**[**^**14**^**C]-Radiolabelled total lipid analysis of *M. smegmatis* strains.** A) Profiles of conditional ∆*pks13* mutant, complemented ∆*pks13* and ∆*pks13* D264A. B) and C) Profiles of a panel of ∆*pks13* strains complemented with SDM (Table 4).

To investigate the impact of the Pks13 site-directed mutations on lipid biosynthesis, metabolic labelling with [^14^C]-acetate was performed using *M. smegmatis* cultures. Under standard conditions, cultures are typically labelled at mid-log phase, OD 600 nm ∼ 0.4, to ensure comparable metabolic activity across samples. However, as the conditional mutant and the D264A strain under non-inducing conditions failed to grow to this density, radiolabelling was initiated at the point of inoculation. This approach ensured sufficient incorporation of [^14^C] acetate and allowed a meaningful comparison of lipid profiles despite severe growth impairment. The lipid analyses focused on trehalose monomycolate (TMM) and trehalose dimycolate (TDM), which serve as key readouts of mycolic acid synthesis and Pks13 activity. As expected, the ∆*pks13* mutant showed a clear dependence on acetamide with TMM and TDM detectable only under +Ac conditions (Fig. 4a). The complemented strain, however, restored TMM and TDM synthesis in both +Ac and —Ac conditions confirming functional complementation (Fig. 4a). In contrast, the D264A mutant displayed a striking defect in TMM and TDM synthesis in the absence of acetamide (Fig. 4a). While TMM and TDM were readily detected under +Ac conditions, both species were substantially reduced under —Ac conditions. This reduction was consistent across replicates and suggests a strong impairment in mycolic acid biosynthesis when the mutant allele is the sole source of Pks13 activity. Importantly, none of the other SDMs showed any noticeable differences in TMM or TDM synthesis in inducing and non-inducing conditions (Fig. 4b, c). Their lipid profiles were comparable to the complemented strain indicating that these mutations do not significantly disrupt Pks13 function under the conditions tested. Taken together, these results show that substitution of D264 uniquely compromises mycolic acid production which is in line with the severe growth defect observed for this mutant. This strongly supports a critical role for this residue in Pks13 function and consequently, in maintaining normal cell envelope lipid composition.

**Fig. 5:**
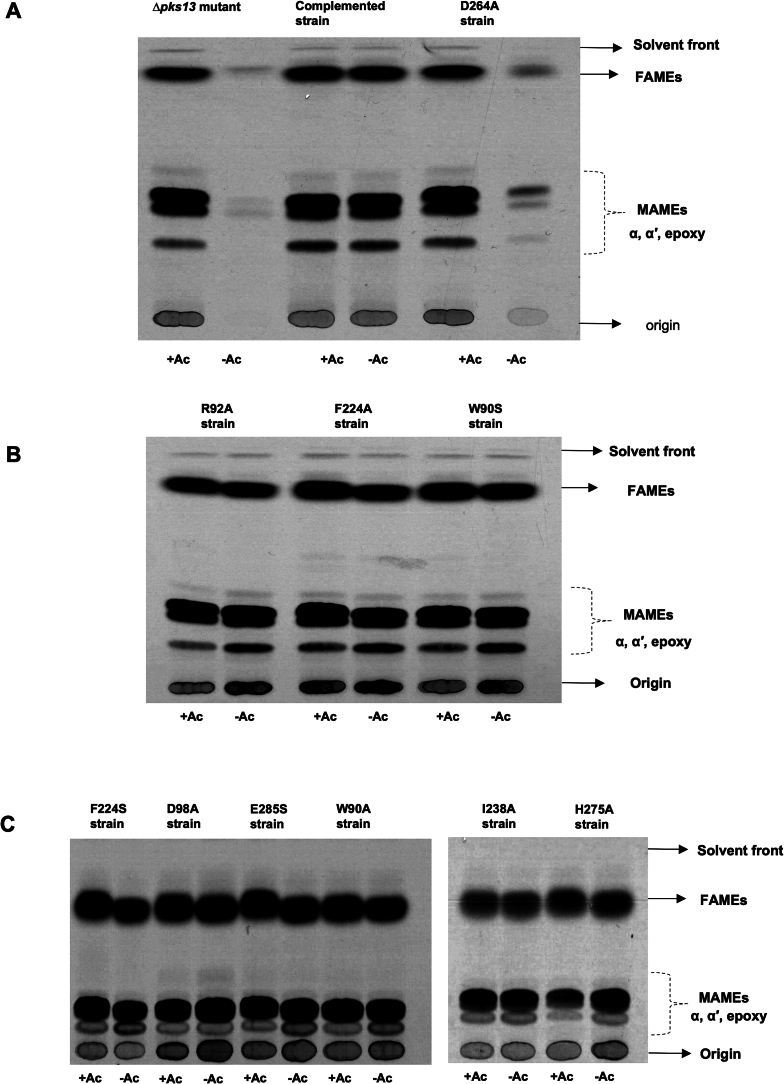
**[**^**14**^**C]-Radiolabelled mycolic acid and fatty acid analysis of *M. smegmatis* strains.** A) Profiles of conditional ∆*pks13* mutant, complemented ∆*pks13* strain, and ∆*pks13* D264A. B) and C) Profiles of a panel of ∆*pks13* strains complemented SDM strains (Table 4).

To assess the effect of the SDMs on total mycolic acid and fatty acid profiles we analyzed mycolic acid methyl esters (MAMEs) and fatty acid methyl esters (FAMES) by thin-layer chromatography (TLC). As expected, (Fig. 5a) the ∆*pks13* mutant showed strong production of FAMEs and MAMEs only in the presence of acetamide conditions confirming the requirement of Pks13 for mycolic acid synthesis. The complemented strain restored production of all major species irrespective of inducer presence, indicating full functional rescue. In contrast, the D264A mutant displayed a pronounced defect in overall mycolic acid synthesis in the absence of acetamide, whilst in the presence of acetamide showed clear bands corresponding to α-, α'- and epoxy-MAMEs, these were markedly reduced in the absence of acetamide. This reduction was not restricted to a specific sub-class of mycolates but was observed across all major species, suggesting a global impairment in mycolic acid biosynthesis. Notably, a concomitant decrease in FAME levels was also observed in the absence of acetamide in the D264A mutant indicating that the defect extends to the broader fatty acid pool. All other SDMs exhibited FAMEs and MAMEs profiles comparable between +Ac and —Ac conditions with no obvious reduction in any of the major mycolic acid species (Fig. 5b, c). Their lipid patterns closely resembled that of the complemented strains suggesting that these substitutions do not significantly impact Pks13 activity or overall lipid homeostasis. Taken together, the data reinforces the conclusion that the D264 residue is critical for Pks13 function. Its substitution leads to a substantial loss of mycolic acid production across all major subclasses accompanied by a reduction in fatty acid synthesis, which likely underpins the severe growth and viability defects observed in this mutant.

## 4 Conclusion

In this study, we show that Asp264 plays a crucial role in maintaining Pks13 function in *M. smegmatis*. Substitution of this residue with alanine results in a severe defect in growth under non-inducing conditions accompanied by a near-complete loss of TMM, TDM, and overall mycolic acid production. A striking observation that this phenotype is unique to the D264A mutant as all other site-directed mutations introduced in and around the KS domain were largely well tolerated with no obvious impact on growth or lipid biosynthesis. This suggests that Asp264 occupies a structurally or functionally essential position within the enzyme rather than contributing in a general or redundant manner to protein stability. Taken together with the structural modelling, our data supports the idea that Asp264 is important for maintaining the correct organization of the KS active-site environment. Although, it is not directly involved in catalysis, the residue sits near the catalytic Cys267 and appears to influence the conformation of a nearby loop that contributes to shaping the substrate-binding pocket. In the D264A mutant, loss of the charged side chain leads to a noticeable local rearrangement in this region including a compaction of the active-site cavity. Given the tight spatial constraints required for proper positioning of long-chain acyl substrates in keto-synthase reactions, even relatively subtle changes in pocket geometry are likely to have significant functional consequences. This provides a plausible explanation for the dramatic reduction in mycolic acid-derived lipids observed in the mutant.

In contrast, the absence of any clear phenotype in the other mutants suggests that the KS domain is relatively robust to peripheral perturbations with only a small number of residues being essential for maintaining catalytic architecture. Most of the substitutions tested are likely located on the surface of the protein or in regions where nearby interactions can compensate for changes, so they do not significantly affect enzyme function under the conditions tested. Asp264 therefore stands out as part of a more sensitive structural network that governs the correct positioning of the catalytic machinery.

Overall, these findings highlight that in addition to the well-known catalytic cysteine, other amino acids in the KS domain can also be very important for enzyme function by modulating local structure rather than directly participating in chemistry. In the case of Asp264, its role appears to be primarily architectural helping to stabilise an active-site configuration that is competent for substrate processing. Given the essential nature of Pks13 in mycolic acid biosynthesis, this residue and its surrounding structural context may represent an interesting point of vulnerability that could be further explored in future studies, particularly in the context of enzyme dynamics and inhibitor design.

## CRediT authorship contribution statement

**Mamata Modak:** Writing – review & editing, Writing – original draft, Methodology, Formal analysis, Conceptualization. **Hemza Ghadbane:** Writing – review & editing, Writing – original draft, Conceptualization. **Klaus Futterer:** Writing – review & editing, Writing – original draft, Conceptualization. **Apoorva Bhatt:** Writing – review & editing, Writing – original draft, Conceptualization. **Gurdyal S. Besra:** Writing – review & editing, Writing – original draft, Supervision, Funding acquisition, Conceptualization.

## Declaration of competing interest

The authors declare the following financial interests/personal relationships which may be considered as potential competing interests: Gurdyal Singh Besra and Apoorva Bhatt report financial support provided by Medical Research Council. Mamata Modak reports financial support provided by Darwin Trust of Edinburgh. Gurdyal Singh Besra also reports a relationship with the Cell Surface as an Editor.

## Data Availability

Data will be made available on request.

## Appendix A Supplementary data

Supplementary material: Supplementary Fig. S1. **RT-PCR analysis confirming transcription of plasmid-borne *M. smegmatis pks13* in complemented strain and site-directed mutant strains**. Top panel, all strains harbouring pMV261- *Ms pks13* including the complemented strain and all site-directed mutants along with wild-type *M. smegmatis* showed robust transcription of *Ms pks13*, confirming plasmid incorporation and expression. The Δ*pks13* conditional mutant showed no *Ms pks13* transcript. Middle panel, *sigA* bands were equivalent across all lanes confirming RNA integrity. Bottom panel, *M. tuberculosis pks13* specific primers amplified a product only from Δ*pks13* conditional mutant, confirming primer specificity.

## References

[bb0005] Bansal-Mutalik R., Nikaido H. (2014). Mycobacterial outer membrane is a lipid bilayer and the inner membrane is unusually rich in diacyl phosphatidylinositol dimannosides. Proc. Natl. Acad. Sci..

[bb0010] Bardarov S., Bardarov S., Pavelka M.S., Sambandamurthy V., Larsen M., Tufariello J., Jacobs W.R. (2002). Specialized transduction: an efficient method for generating marked and unmarked targeted gene disruptions in Mycobacterium tuberculosis, M. Bovis BCG and M. Smegmatis. Microbiology.

[bb0015] Barry C.E., Lee R.E., Mdluli K., Sampson A.E., Schroeder B.G., Slayden R.A., Yuan Y. (1998). Mycolic acids: structure, biosynthesis and physiological functions. Prog. Lipid Res..

[bb0020] Bon C., Cabantous S., Julien S., Guillet V., Chalut C., Rima J., Mourey L. (2022). Solution structure of the type I polyketide synthase Pks13 from Mycobacterium tuberculosis. BMC Biol..

[bb0025] Butler W.R., Jost K.C., Kilburn J.O. (1991). Identification of mycobacteria by high-performance liquid chromatography. J. Clin. Microbiol..

[bb0030] Daffé M., Draper P. (1997). The envelope layers of mycobacteria with reference to their pathogenicity. Adv. Microb. Physiol..

[bb0035] Daffé M., Marrakchi H. (2019). Unraveling the structure of the mycobacterial envelope. Microbiol. Spectrum.

[bb0040] D’Ambrosio H.K., Ganley J.G., Keeler A.M., Derbyshire E.R. (2022). A single amino acid residue controls acyltransferase activity in a polyketide synthase from Toxoplasma gondii. iScience.

[bb0045] Daugelat S., Kowall J., Mattow J., Bumann D., Winter R., Hurwitz R., Kaufmann S.H. (2003). The RD1 proteins of Mycobacterium tuberculosis: expression in Mycobacterium smegmatis and biochemical characterization. Microbes Infect..

[bb0050] Dobson G., Minnikin D.E., Minnikin S.M., Parlett J.H., Goodfellow M., Ridell M., Goodfellow M., Minnikin D.E. (1985). Chemical Methods in Bacterial Systematics.

[bb0055] Dubnau E., Chan J., Raynaud C., Mohan V.P., Lanéelle M.A., Yu K., Daffé M. (2000). Oxygenated mycolic acids are necessary for virulence of Mycobacterium tuberculosis in mice. Mol. Microbiol..

[bb0060] Gande R., Gibson K.J., Brown A.K., Krumbach K., Dover L.G., Sahm H., Eggeling L. (2004). Acyl-CoA carboxylases (accD2 and accD3), together with a unique polyketide synthase (cg-pks), are key to mycolic acid biosynthesis in Corynebacterianeae such as Corynebacterium glutamicum and Mycobacterium tuberculosis. J. Biol. Chem..

[bb0065] Garton N.J., Christensen H., Minnikin D.E., Adegbola R.A., Barer M.R. (2002). Intracellular lipophilic inclusions of mycobacteria *in vitro* and *in sputum*. Microbiology.

[bb0070] Gavalda S., Léger M., van Der Rest B., Stella A., Bardou F., Montrozier H., Quemard A. (2009). The Pks13/FadD32 crosstalk for the biosynthesis of mycolic acids in Mycobacterium tuberculosis. J. Biol. Chem..

[bb0075] Gavalda S., Bardou F., Laval F., Bon C., Malaga W., Chalut C., Quémard A. (2014). The polyketide synthase Pks13 catalyzes a novel mechanism of lipid transfer in mycobacteria. Chem. Biol..

[bb0080] Hart E.M., Bernhardt T.G. (2025). The mycomembrane. Curr. Biol..

[bb0085] Jackson M. (2014). The mycobacterial cell envelope—lipids. Cold Spring Harb. Perspect. Med..

[bb0090] Jain P., Hsu T., Arai M., Biermann K., Thaler D.S., Nguyen A., Jacobs W.R. (2014). Specialized transduction designed for precise high-throughput unmarked deletions in Mycobacterium tuberculosis. MBio.

[bb0095] Johnston H.E., Batt S.M., Brown A.K., Savva C.G., Besra G.S., Fütterer K. (2024). Cryo-electron microscopy structure of the di-domain core of Mycobacterium tuberculosis polyketide synthase 13, essential for mycobacterial mycolic acid synthesis. Microbiology.

[bb0100] Kim S.K., Dickinson M.S., Finer-Moore J., Guan Z., Kaake R.M., Echeverria I., Stroud R.M. (2023). Structure and dynamics of the essential endogenous mycobacterial polyketide synthase Pks13. Nat. Struct. Mol. Biol..

[bb0105] Minnikin D.E., Minnikin S.M., Goodfellow M. (1982). The oxygenated mycolic acids of Mycobacterium fortuitum, M. Farcinogenes and M. Senegalense. Biochim. Biophys. Acta.

[bb0110] Murry J., Sassetti C.M., Moreira J., Lane J., Rubin E.J. (2005). A new site-specific integration system for mycobacteria. Tuberculosis.

[bb0115] Ojha A., Anand M., Bhatt A., Kremer L., Jacobs W.R., Hatfull G.F. (2005). GroEL1: a dedicated chaperone involved in mycolic acid biosynthesis during biofilm formation in mycobacteria. Cell.

[bb0120] Parish T. (2021). Mycobacteria protocols.

[bb0125] Portevin D., de Sousa-D'Auria C., Houssin C., Grimaldi C., Chami M., Daffé M., Guilhot C. (2004). A polyketide synthase catalyzes the last condensation step of mycolic acid biosynthesis in mycobacteria and related organisms. Proc. Natl. Acad. Sci..

[bb0130] Portevin D., de Sousa-D’Auria C., Montrozier H., Houssin C., Stella A., Lanéelle M.A., Daffé M. (2005). The acyl-AMP ligase FadD32 and AccD4-containing acyl-CoA carboxylase are required for the synthesis of mycolic acids and essential for mycobacterial growth: identification of the carboxylation product and determination of the acyl-CoA carboxylase components. J. Biol. Chem..

[bb0135] Robbins T., Kapilivsky J., Cane D.E., Khosla C. (2016). Roles of conserved active site residues in the ketosynthase domain of an assembly line polyketide synthase. Biochemistry.

[bb0140] Stover C.K., De La Cruz V.F., Fuerst T.R., Burlein J.E., Benson L.A., Bennett L.T., Bloom B.R. (1991). New use of BCG for recombinant vaccines. Nature.

[bb0145] Vander Beken S., Al Dulayymi J.A.R., Naessens T., Koza G., Maza-Iglesias M., Rowles R., Grooten J. (2011). Molecular structure of the Mycobacterium tuberculosis virulence factor, mycolic acid, determines the elicited inflammatory pattern. Eur. J. Immunol..

[bb0150] Xia F., Zhang H., Yang H., Zheng M., Min W., Sun C., Yang P. (2023). Targeting polyketide synthase 13 for the treatment of tuberculosis. Eur. J. Med. Chem..

[bb0155] Yamaryo-Botte Y., Rainczuk A.K., Lea-Smith D.J., Brammananth R., van der Peet P.L., Meikle P., McConville M.J. (2015). Acetylation of trehalose mycolates is required for efficient MmpL-mediated membrane transport in Corynebacterineae. ACS Chem. Biol..

