# Supplementary figures and images for: Functional importance of Asp264 in the ketosynthase domain of Pks13 in Mycobacterium smegmatis
Source: Cell Surf. 2026 Jun 24;16:100178. doi: 10.1016/j.tcsw.2026.100178 (PMC13324839; doi:10.1016/j.tcsw.2026.100178)

Supplementary Fig 1

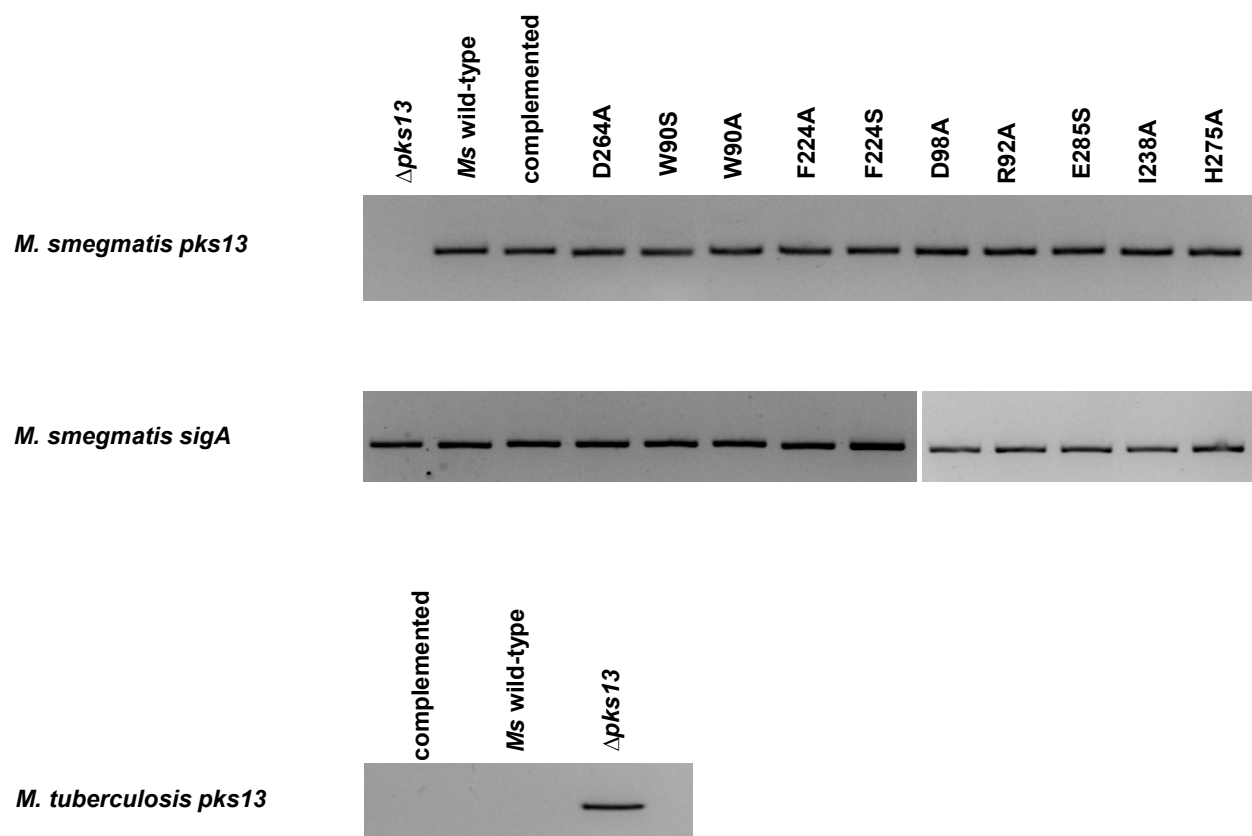

Supplement: Supplementary file 1 — Supplementary material: Supplementary Fig. S1. RT-PCR analysis confirming transcription of plasmid-borne M. smegmatis pks13 in complemented strain and site-directed mutant strains. Top panel, all strains harbouring pMV261- Ms pks13 including the complemented strain and all site-directed mutants along with wild-type M. smegmatis showed robust transcription of Ms pks13, confirming plasmid incorporation and expression. The Δpks13 conditional mutant showed no Ms pks13 transcript. Middle panel, sigA bands were equivalent across all lanes confirming RNA integrity. Bottom panel, M. tuberculosis pks13 specific primers amplified a product only from Δpks13 conditional mutant, confirming primer specificity. [file mmc1.pdf]
